# Use of Internet Search Queries to Enhance Surveillance of Foodborne Illness

**DOI:** 10.3201/eid2111.141834

**Published:** 2015-11

**Authors:** Gyung Jin Bahk, Yong Soo Kim, Myoung Su Park

**Affiliations:** Kunsan National University, Gunsan, South Korea (G.J. Bahk, M.S. Park);; Korea Health Industry Development Institute, Cheongwon, South Korea (Y.S. Kim)

**Keywords:** foodborne illness, Internet search query, Internet-based surveillance, time-series analysis, SARIMA model, surveillance, enteric infections, bacteria

## Abstract

As a supplement to or extension of methods used to determine trends in foodborne illness over time, we propose the use of Internet search metrics. We compared Internet query data for foodborne illness syndrome–related search terms from the most popular 5 Korean search engines using Health Insurance Review and Assessment Service inpatient stay data for 26 International Classification of Diseases, Tenth Revision, codes for foodborne illness in South Korea during 2010–2012. We used time-series analysis with Seasonal Autoregressive Integrated Moving Average (SARIMA) models. Internet search queries for “food poisoning” correlated most strongly with foodborne illness data (*r* = 0.70, p<0.001); furthermore, “food poisoning” queries correlated most strongly with the total number of inpatient stays related to foodborne illness during the next month (β = 0.069, SE 0.017, p<0.001). This approach, using the SARIMA model, could be used to effectively measure trends over time to enhance surveillance of foodborne illness in South Korea.

## Abstract 2

“Food poisoning” queries were correlated with the number of foodborne illness–related hospital stays.

Foodborne illness is a growing public health problem in developing and industrialized nations and a common cause of illness, and sometimes death, worldwide (*1*). However, exact morbidity associated with foodborne illnesses is difficult to determine because many cases of foodborne illness are underdiagnosed or underreported and thus not identified by public health surveillance systems (*2*).

The objective of public health surveillance systems for foodborne illnesses is to identify the causes of foodborne disease so that prevention and control programs can be introduced and, if necessary, strengthened (*3*). The overall quality or validity of a public health surveillance system depends on the quality of the data in terms of the following 3 factors: completeness, timeliness, and consistency (*4*,*5*). To estimate the effect of an illness on a person’s overall health, specific information about incidence is required, along with the development of a method for estimating the completeness of reporting. Furthermore, detection of outbreaks necessitates comparison of current reporting with the expected baseline, and timeliness is highly relevant. Finally, to measure trends over time, reporting must be kept consistent, so that the techniques used to detect underlying factors do not change (*4*,*6*,*7*).

M’ikanatha et al. (*8*) and Vogt et al. (*9*) suggested that the benefits of electronic and Web‐based reporting systems for infectious disease surveillance data include improved timeliness and completeness. Internet-based public health surveillance is a new approach that can be performed by using syndrome- and disease-specific terms (*10*,*11*). The relative frequency of certain Internet queries is highly correlated with the occurrence of some infectious disease symptoms (*10*–*12*). Internet-based surveillance systems offer a new and developing means of measuring trends over time (consistency) and monitoring the effectiveness of various public health concern interventions, including those for emerging infectious diseases (*13*). To enhance the consistency in public health surveillance systems for foodborne illness, we propose the use of Internet search query data.

Internet availability and use has increased greatly during the past 10 years (*14*). The availability of health-related information on the Internet has changed how persons seek information about health (*15*). Although Internet-based surveillance systems do not have the capacity to completely replace traditional surveillance systems (*16*), they do provide a new means by which to detect and monitor infectious diseases. In a study reviewing Internet-based surveillance systems, Milinovich et al. (*16*) suggested that future research in this area should focus on using data generated through Internet-based surveillance and response systems to bolster the capacity of traditional surveillance systems for emerging infectious diseases.

Pelat et al. (*17*) emphasized the need for query surveillance studies on diseases other than influenza or in languages other than English. Recently, Desai et al. (*10*) compared norovirus outbreak surveillance data with Google Internet query data. Wilson and Brownstein (*18*) performed search-term surveillance of a listeriosis outbreak in Canada. The results of these studies suggest that Internet surveillance tools can assist in the early identification of foodborne disease outbreaks. However, these 2 studies are among relatively few that have addressed possible relationships between Web search queries and foodborne illness, especially bacterial foodborne illness.

In South Korea, the Health Insurance Review and Assessment Service (HIRA) (*19*) reviews medical fees and evaluates the appropriateness of medical benefits provided to patients. For this purpose, HIRA data have been gathered for all patients in South Korea. These data include foodborne illness and many other infectious diseases and can be used for public health surveillance. Furthermore, by comparing the data generated by the HIRA surveillance system with that collected from Internet search queries, a more comprehensive surveillance system can be created. This combined surveillance system could contribute to the strength of traditional surveillance systems for foodborne illness in South Korea.

To assess whether Internet search query trends can be used to effectively measure trends over time in the spread of foodborne illnesses, particularly those caused by bacteria, we compared Internet query data for 5 foodborne illness syndrome–related search terms from the most popular 5 search engines in South Korea with HIRA data in South Korea. We used time-series analysis, taking into account lagged effects, autocorrelation, and the seasonal fluctuation in incidences of foodborne illness.

## Methods

### Data on Bacterial Foodborne Illness

We included data about bacterial foodborne illness and infectious enteritis (i.e., acute gastroenteritis) because some bacterial infectious enteritis symptoms are similar to those of bacterial foodborne illness. Foodborne illnesses caused by viruses, protozoa, natural toxins, and chemical agents were excluded because these fell outside the scope of the current study. We collected data on bacterial foodborne illness from HIRA (*19*) for 2010–2012 using a method of Park et al. (*20*). From the total set of patient data, we extracted cases in which bacterial foodborne diseases and intestinal infections had been diagnosed by using the Korean Standard Classification of Diseases (*21*). This classification is based on, and highly similar to, the International Classification of Diseases, Tenth Revision (ICD-10), issued by the World Health Organization but is adapted for use in South Korea. ICD-10 assigns numeric codes to specific illnesses to standardize diagnosis for epidemiology, health management, and clinical purposes (*22*). The 26 ICD-10 codes defining bacterial foodborne illness and infectious enteritis comprise diagnosis codes in the following range: A02.0, A02.8–9, A03.0–3, A03.8–9, A04.0–6, A04.8–9, A05.0–4, A04.8–9, and A32 (Table 1). We included cases that corresponded to these codes and classified them accordingly. Then we grouped cases according to whether they resulted in inpatient stays or outpatient visits and the month and year in which they occurred. Because preanalyses showed a stronger correlation between Internet search queries and HIRA inpatient stays than between Internet search queries and outpatient visits or officially reported data, we used only HIRA inpatient data for the analysis.

**Table 1:** ICD-10 codes for causes of bacterial foodborne illness and infectious enteritis and number of inpatient hospital stays for each, South Korea, January 2010–December 2012*

ICD-10 code	Diagnosis	No. inpatient stays		
		All 3 y	3-y monthly average ± SD	%
A02	Nontyphoidal *Salmonella* infections			
A02.0	*Salmonella* Enteritis (salmonellosis)	1,623	45.1 ± 21.3	4.8
A02.8	Other specified salmonella infections	100	2.8 ± 3.1	0.3
A02.9	Salmonella infection, unspecified	1,251	34.8 ± 17.5	3.7
A03	Shigellosis			
A03.0	Shigellosis due to *Shigella dysenteriae*	13	1.3 ± 0.7	0.1
A03.1	Shigellosis due to *Shigella flexneri*	54	2.1 ± 1.5	0.2
A03.2	Shigellosis due to *Shigella boydii*	7	1.2 ± 0.4	0.1
A03.3	Shigellosis due to *Shigella sonnei*	50	2.3 ± 1.2	0.2
A03.8	Other shigellosis	20	1.5 ± 1.1	0.2
A03.9	Shigellosis, unspecified	190	5.4 ± 4.4	0.6
A04	Other bacterial intestinal infections			
A04.0	Enteropathogenic *Escherichia coli* infection	131	3.6 ± 2.8	0.4
A04.1	Enterotoxigenic *Escherichia coli* infection	13	1.2 ± 0.6	0.1
A04.2	Enteroinvasive *Escherichia coli* infection	7	1.2 ± 0.4	0.1
A04.3	Enterohemorrhagic *Escherichia coli* infection	72	2.6 ± 1.9	0.3
A04.4	Other intestinal *Escherichia coli* infection	497	13.8 ± 4.6	1.5
A04.5	*Campylobacter* enteritis	39	2.0 ± 1.1	0.2
A04.6	Enteritis due to *Yersinia enterocolitica*	6	1.2 ± 0.4	0.1
A04.8	Other specified bacterial intestinal infections	3.453	95.9 ± 28.0	10.2
A04.9	Bacterial intestinal infection, unspecified	20,897	580.5 ± 114.1	62.0
A05	Other bacterial foodborne intoxications, not elsewhere classified			
A05.0	Foodborne staphylococcal intoxication	42	2.0 ± 1.2	0.2
A05.1	Botulism (classical foodborne intoxication due to *Clostridium botulinum*)	11	1.4 ± 0.7	0.1
A05.2	Foodborne *Clostridium perfringens* (*Clostridium welchii*) intoxication	60	2.2 ± 1.2	0.2
A05.3	Foodborne *Vibrio parahaemolyticus* intoxication	132	5.5 ± 6.5	0.6
A05.4	Foodborne *Bacillus cereus* intoxication	14	2.8 ± 2.7	0.3
A05.8	Other specified bacterial foodborne intoxications	348	9.7 ± 4.8	1.0
A05.9	Bacterial foodborne intoxication, unspecified	4,069	113.0 ± 40.6	12.1
A32	Listerial foodborne infection	30	1.5 ± 0.8	0.2
Total		33,129	936.4 ± 190.2	100.0

*ICD-10, International Classification of Diseases, Tenth Revision.

### Internet Query Data

We analyzed Internet queries submitted to the 5 most popular Internet search websites in South Korea: Naver (http://www.naver.com), Daum (http://www.daum.net), Google (http://www.google.co.kr), Nate (http://www.nate.com), and Yahoo! Korea (http://www.yahoo.co.kr). These websites are written in Korean; the submitted queries were also in Korean. The national market share of these websites during the analysis period (January 2010–December 2012) was 68.2%, 21.1%, 5.3%, 2.7%, and 1.5%, respectively, totaling 98.8% of the Internet search market (*23*). However, we did not compare metrics from different search engines because the most popular 2 occupied 89.4% of the market. According to the Korea Internet and Security Agency (*24*), 72.3% of the Korean population uses the Internet daily. The query data were aggregated nationally.

Although more serious complications and other problems can result from foodborne illness, foodborne illness can be defined as any form of infectious gastroenteritis caused by eating food, including food contaminated immediately before ingestion (*25*,*26*). Thus, we chose to focus on 3 common symptoms of bacterial foodborne illness and infectious enteritis: diarrhea, vomiting, and abdominal pain after food consumption that could not be attributed to other factors, such as advanced pregnancy, drug use, and/or alcohol consumption. In addition, we referenced the Standard Korean Dictionary (*27*) to source a representative keyword that Koreans use to define foodborne diseases. Finally, the queries included the following 5 terms related to foodborne illness (Korean translations in parentheses): “food poisoning” (Sik-jung-dog), “diarrhea” (Seol-sa), “vomiting” (Gu-tto), “abdominal pain” (Bok-tong), and “gastroenteritis” (Jang-yeum). We collected monthly data on these 5 Internet search query terms from January 2010 through December 2012 because the HIRA data on bacterial foodborne illness are also available monthly. The data collection procedure was conducted by WISEnut Korea (Seongnam, South Korea; http://www.wisenut.co.kr), a company specializing in the collection and analysis of large datasets, by using a Korea-dedicated Web crawler to access the 5 most popular Internet search websites in South Korea.

### Data Analysis

The collected Internet query data for South Korea were aggregated for each month. To quantify the strength of associations between incidences of foodborne illness and each search term, we calculated the Spearman *r* correlation, taking into account lead or lag effects, with the variables temporally leading and lagged by up to 2 months. For better prediction, the seasonal autoregressive integrated moving average (SARIMA) model was used to estimate the parameters of the regression model through the preprocessing of a stationary time series. The SARIMA model is used for time-series modeling and forecasting and is based on Box and Jenkins’ ground-breaking work, which takes into account the impact of seasonality and autocorrelations on the variables (*28*,*29*). A SARIMA model can be described as an ARIMA (p, d, q) multiplied by (P, D, Q), wherein p, d, q represent ordinary components and P, D, Q represent seasonal components and p is the number of autoregressive terms, d is the number of nonseasonal differences needed for stationarity, q is the number of lagged forecast errors in the prediction equation, P is the number of seasonal autoregressive terms, D is the number of seasonal differences, and Q is the number of seasonal moving average terms. These terms or numbers were determined through the autocorrelation function and the partial autocorrelation function. The Akaike Information Criterion was used to assist the model fits, and the residuals were further examined for autocorrelation by plotting scatter diagrams, as well as the autocorrelation function and partial autocorrelation function (*30*). These processes were conducted with SPSS software (SPSS Inc., Chicago, IL, USA). All the analyses were performed using IBM SPSS version 21.0 (Data Solution Inc., Seoul, South Korea) with a significance level of p = 0.05.

## Results

During 2010–2012, a total of 33,129 inpatient hospital stays involved diagnoses of bacterial foodborne illness and infectious enteritis (corresponding to 1 of the ICD-10 codes; Table 1). This number represents an average of 936 ± 190 hospital stays per month during the 3-year study period, or 22 inpatient stays per 100,000 persons in South Korea annually. Nearly 62.0% of patients in surveyed inpatient stays had diagnoses of unspecified bacterial intestinal infection (A04.9), and 12.1% had diagnoses of unspecified bacterial foodborne intoxication (A05.9). Of specific diagnoses, *Salmonella* (A02.0, A02.8−9) was the most common (8.8% of patients), followed by pathogenic *Escherichia coli* (A04.0−4) (2.4%) (Table 1).

During the 3 years examined, 2,943,776 queries containing at least 1 of the 5 foodborne illness–related search terms included in this study were submitted to the most popular 5 Internet search engines in South Korea. Of these, diarrhea was the most frequent (found in 1,401,515 [47.6%] searches), followed by gastroenteritis (574,389 [19.5%]), vomiting (403,406 [13.7%]), food poisoning (293,860 [10.0%]), and abdominal pain (270,606 [9.2%]).

Of the 5 search terms, the prevalence of searches for food poisoning correlated most strongly with the number of inpatient stays related to bacterial foodborne illness and infectious enteritis for all surveyed ICD-10 codes (*r* = 0.68, p<0.001) (Table 2). Although diarrhea was the most frequently searched of all the terms, its correlation with total hospital stays for all surveyed conditions (*r* = 0.48, p = 0.003) was weaker than for food poisoning or gastroenteritis (*r* = 0.52, p = 0.001). Abdominal pain (*r* = 0.38, p<0.022) and vomiting (*r* = 0.34, p = 0.040) showed the weakest correlations with total hospital stays for all surveyed conditions.

**Table 2:** Spearman *r* correlation between number of inpatient hospital stays for types of bacterial foodborne illness and infectious enteritis and number of Internet searches for food poisoning with lead and lag times of up to 2 mo, South Korea, January 2010–December 2012*

Diagnosis (ICD-10 code)	Previous 2 months	Previous 1 month	Same month	Following 1 month	Following 2 months
Salmonellosis (A02.0, A02.8−9)	−0.173	0.218	0.546†	0.629†	0.618†
Campylobacteriosis (A04.5)	−0.200	0.298	0.523†	0.545†	0.366‡
Other bacterial intestinal infections (A04.8−9)	−0.126	0.211	0.587†	0.671†	0.535†
Other bacterial foodborne intoxications (A05.8−9)	−0.080	0.268	0.678†	0.641†	0.395‡
Total bacterial foodborne illness and infectious enteritis (all of the codes in Table 1)	−0.112	0.254	0.679†	0.701†	0.545†

*ICD-10, International Classification of Diseases, Tenth Revision. †p<0.01. ‡p<0.05.

**Figure 1:**
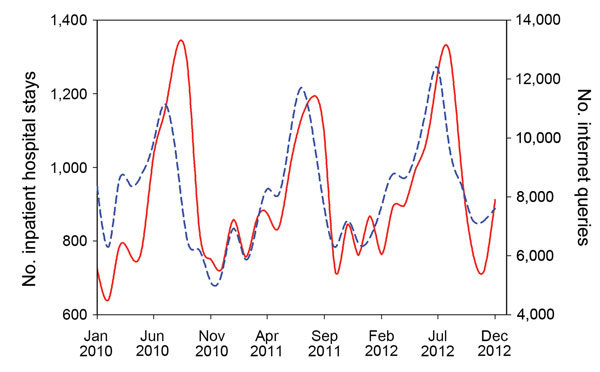
Number of Internet search queries for food poisoning (short dashed blue line) and estimated number of inpatient hospital stays for bacterial foodborne illness and infectious enteritis (solid red line), South Korea, January 2010–December 2012.

In most cases, the number of Internet search queries for a term was high in 1 month and then the next month the number of related hospital stays was high (Figure 1; Table 2). Searches for food poisoning correlated most strongly with inpatient stays for diagnostic code A04.8−9 (other bacterial intestinal infections) in the next month (*r* = 0.67, p<0.001). However, these terms correlated even more strongly with the total number of hospital stays for all surveyed conditions in the next month (*r* = 0.70, p<0.001). For all specified pathogens, hospital stays related to *Salmonella* (A02.0, A02.8−9) correlated most strongly with Internet searches for food poisoning in the previous month (*r* = 0.63, p<0.001). Except for *Campylobacter* (A04.5), bacterial foodborne pathogens were weakly or not correlated (p>0.05) with most search queries (Table 2). Internet searches for terms included in the study from the 2 months before and 2 months after were more weakly (in some cases negatively) correlated with hospital stays for all conditions. Internet searches for food poisoning occurred 1−2 months before the inpatient hospital stays (Figure 1), which suggests the possibility of a strongly lagged relationship.

**Figure 2:**
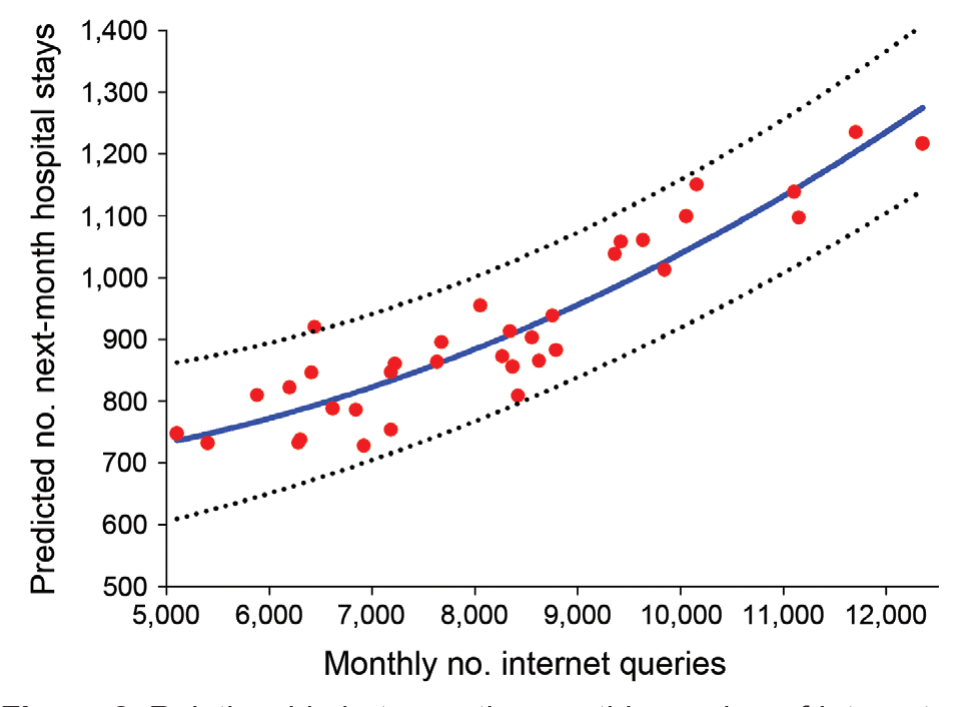
Relationship between the monthly number of Internet search queries for food poisoning and predicted number of inpatient hospital stays for total bacterial foodborne illness and infectious enteritis for next month by seasonal autoregressive integrated moving average model, South Korea, January 2010–December 2012. Red dots and blue line represent actual and predicted numbers of inpatient hospital stays, respectively. Dotted lines indicate 95% CIs (R^2^ = 0.71).

The best regression model that had the highest correlation value for Internet searches relating to food poisoning and the total number of hospital stays for all conditions surveyed in the next month was SARIMA (1, 0, 0) (1, 0, 0)_12_ with Akaike Information Criterion 433.6; that is, first-order (seasonal) autoregressive model (p and P = 1, respectively). The parameters estimated by the best SARIMA model are shown in Table 3. The significant parameters in the model include first-order autoregression of the number of inpatient hospital stays and seasonal autoregression, as well as the Internet search query “food poisoning” 1 month earlier (β = 0.045, SE 0.017, p<0.05). With regard to goodness of fit, residuals were randomly distributed with no autocorrelation among them. The incidence of the food poisoning query was positively associated with the number of inpatient hospital stays for total bacterial foodborne illness and infectious enteritis for the next month (Figure 2). This association that Internet search queries can be used to track trends over time in relation to foodborne illness.

**Table 3:** Parameters estimated by the seasonal autoregressive integrated moving average (1,0,0)(1,0,0) model regarding effects of Internet searches for food poisoning on number of inpatient hospital stays for total bacterial foodborne illness and infectious enteritis in the next month, South Korea, January 2010–December 2012

Variable	β	SE	p value
First-order autoregression	0.4059	0.1546	0.0133
First-order seasonal autoregression	0.5715	0.1447	0.0004
Food poisoning queries 1 mo. earlier	0.0450	0.0176	0.0157
Constant	557.1300	152.6156	0.0010


## Discussion

We assessed relationships between Internet query data for foodborne illness syndrome–related search terms and inpatient hospital stays in which bacterial foodborne illness and infectious enteritis were diagnosed in South Korea. The search query data in the month before hospital stay can be used as early indicators to measure trends over time in foodborne illness in South Korea.

Effective initiation of public health intervention measures depends on early and rapid identification of infectious disease outbreaks (*19*). Early detection of disease activity after a rapid response can reduce the effect of the disease on the general public and is one way to improve early detection monitoring health-seeking behavior in the form of queries entered into Internet search engines (*11*). Ginsberg et al. (*11*) investigated how Google search queries correlated with reports of an influenza epidemic, and Polgreen et al. (*12*) used a Yahoo! query log to investigate the same topic; Hulth et al. (*13*) used the query log of a Switzerland-based Web search engine. This approach obtained more data than did traditional disease surveillance (*19*).

Most existing surveillance systems for foodborne illness are based on disease reporting or on laboratory-based surveillance, which also provide crucial information for assessing foodborne disease trends and enable assessment of data trends over time (*31*) but are passive and record only a minor proportion of all cases in the population (*32*). To estimate the true incidence of and monitor trends over time in foodborne illness, population-based prospective studies as active surveillance have been conducted, such as FoodNet in the United States (*33*,*34*) and OzFoodnet in Australia (*35*), but these population studies are expensive and time consuming (*32*). Data generated through Internet-based surveillance can be used to strengthen the capacity of traditional disease surveillance systems (*16*) in foodborne illness.

In determining trends over time in foodborne illness, Internet search queries will greatly aid existing systems. Public health surveillance systems are under development. By monitoring trends in the incidence and proportion of different types of foodborne illness over time, these Internet-based surveillance systems will provide critical information for evaluating the impact of measures to prevent foodborne illness. Consistent Internet-based surveillance systems with an early warning function will thus benefit the development of future foodborne disease prevention measures.

Numerous studies have used online health-seeking behavior to monitor disease incidence by using various methods, for example, correlation analysis or regression modeling. In this study, we used SARIMA models to analyze incidences of inpatient stays in relation to Internet search queries. SARIMA modeling is a statistical approach used to model and forecast nonstationary time series and instances wherein observations are seasonally dependent and autocorrelated (*28*). SARIMA models have previously been used to quantify the relationship between infectious diseases and other variables (*36*,*37*). However, the SARIMA model used in this study can be applied only minimally in relation to the effect of Internet queries on enteric infection. The SARIMA model we developed did not show a perfect goodness of fit because of the unavailability of data; however, results indicate that it could effectively quantify the relationship between data relating to Internet queries and enteric infections, including foodborne illness.

Of the 5 Korean search terms included, Internet searches for food poisoning were the most effective in predicting inpatient stays. However, we used only 5 syndrome-related search terms because our study was designed to assess a general tendency regarding the relationship between Internet searches and the changing rates of foodborne illness. Flint et al. (*38*) suggested that many episodes of foodborne illness are marked by acute gastroenteritis; however, because not all cases of acute gastroenteritis are caused by organisms found in food, gastrointestinal symptoms do not necessarily indicate a foodborne illness. Thus, if more keywords were selected to reflect terms most likely associated with foodborne illness symptoms, and a filtering procedure was conducted, correspondence between Internet searches and hospitalizations for specific conditions could be closer. Moreover, temporal associations between foodborne illnesses caused by specific pathogens and Internet query data related to symptoms should be studied further.

We used HIRA data on the number of inpatient hospital stays in which bacterial foodborne illness and infectious enteritis were diagnosed, rather than officially reported foodborne disease data. Officially reported foodborne disease data probably fail to capture a substantial proportion of foodborne illness because only cases that are identified and reported are included (*39*). However, South Korea has implemented a mandatory health insurance system managed by HIRA. Therefore, HIRA data represent the total patient population in South Korea, including those who have had foodborne illness. Consequently, the number of inpatient hospital stays, as indicated in the HIRA data, more accurately indicates the trend or prevalence of foodborne illness, which was the focus of this study.

Some weaknesses are associated with the HIRA data. Because the data source we selected is for inpatient hospital stays in which bacterial foodborne illness and infectious enteritis was diagnosed, we do not know what proportion represents cases in which the patient’s illness was actually caused by food. The proportion of pathogen-specific illnesses resulting from eating contaminated food is difficult to accurately estimate (*40*). ICD-10 code A05 specifically refers to foodborne illness, so we can be reasonably confident that most hospital stays in which this diagnosis was made represent actual cases of foodborne illness. However, the proportion of cases in which other diagnoses were made (A02−4) represents illnesses transmitted by food is unclear. Because of these limitations, the actual number of inpatient hospital stays related to foodborne illness for each month might differ from those on which we based our analysis. However, we believe that estimates obtained in this way will be related to Internet query data in a similar fashion to the true values for rates of foodborne illness during the same period. On the basis of this belief, we showed that Internet query data can predict rates of bacterial foodborne illness over time.

Internet-based surveillance systems should not be viewed as an alternative to traditional surveillance systems but rather an extension; therefore, future research needs to focus on how to use Internet-based surveillance systems to complement existing systems (*12*,*13*,*16*). Researchers should preferably validate data from Internet surveillance systems against a body of real events and develop methods that can be used for this purpose—for example, by comparison with national surveillance case data or against data on foodborne outbreaks reported to public health authorities during the same period.

In conclusion, our results showed that search query data can be used to predict changes in the incidence of bacterial foodborne illness over time to a large extent with time-series analysis (SARIMA model). According to the Korea Internet & Security Agency (*24*), the rate of Internet use was 82.1% in South Korea in 2013, compared with 65.5% in 2003. These data suggest that Internet use is increasing substantially and is likely to continue to increase. Therefore, use of Internet search data to predict the incidence of foodborne illness will become a more viable approach and could help to develop a stable and consistent platform to assist foodborne illness surveillance in South Korea.
